# Red Algae From Insular Environments in the Mexican Atlantic: Taxonomic Diversity, Conservation, and Biogeographic Affinities

**DOI:** 10.1002/ece3.72919

**Published:** 2026-03-17

**Authors:** Martha Isabel Vilchis, Oscar E. Hernández, María Luisa Núñez Resendiz, Kurt M. Dreckmann, Ileana Ortegón‐Aznar, Abel Sentíes

**Affiliations:** ^1^ Departamento de Botánica Universidad Autónoma de Yucatán Mérida México; ^2^ Doctorado en Ciencias Biológicas y de la Salud Universidad Autónoma Metropolitana Iztapalapa (UAM‐I) Ciudad de México México; ^3^ Departamento de Hidrobiología Universidad Autónoma Metropolitana‐Iztapalapa Ciudad de México México

**Keywords:** complementary analysis, disjunct distribution, insular phycoflora, island, Mexico, reef systems

## Abstract

One of the most representative groups of the Mexican insular environments is the red algae; although extensive information exists on the insular phycoflora of Mexico, there is no description of the distribution of the group's taxonomic diversity, and few studies address it from a conservation perspective or discuss its biogeographical affinities. In this study, we address these three aspects of red algae along the Mexican Atlantic coast. We recorded 345 species classified into 22 orders, 50 families, and 148 genera, all distributed in 32 insular structures. Cozumel Island, Mujeres Island, and Alacranes reef were the insular structures with the highest taxonomic diversity. The low values of floristic similarity could be related to the high habitat heterogeneity characteristic of the area. Our complementarity values indicate that protecting Cozumel Island and Anegada de Adentro reef would conserve approximately 98% of the insular red algae present in the Mexican Atlantic. 70% of the recorded species have a disjunct distribution range, while the remaining 30% have a restricted distribution range. Continued conservation efforts for these organisms are recommended, with the objective of supporting their eventual incorporation into Mexican environmental protection frameworks.

## Graphical abstract

In this study, we present the first analysis of diversity and distribution of red algae from insular environments in the Mexican Atlantic, with the aim to identify and describe their patterns of taxonomic diversity, to propose priority areas for conservation, and show their biogeographical affinities.

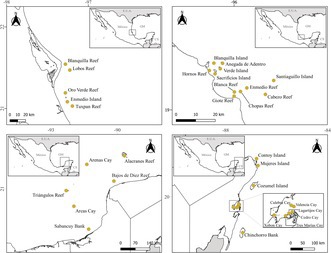

## 1 Introduction

Insular environments are geographical areas clearly differentiated from the environment that surrounds them; a classic example is that of oceanic islands, which are defined as a portion of land surrounded by water. Other examples that can be found in literature are reefs, lakes, or mountain peaks (sometimes considered as insular subcategories) (Drake et al. 2002; INEGI 2015; Vilchis et al. 2024), whose isolation, size, and origin allow their ecological processes and evolutionary dynamics to be very particular, leading to the development of many theories and hypotheses within ecology, evolution, and biogeography (MacArthur and Wilson 1967; Whittaker 1998; Watson 2002; Whittaker and Fernández‐Palacios 2007; Pozo and Llorente‐Bousquets 2001; Vilchis et al. 2024). In terms of conservation, island environments have become the focus of attention for specialists in this area, since they are among the most biodiverse ecosystems in the world, many of them endemic, and whose populations are highly vulnerable to natural disasters, anthropogenic activities, global climate changes, and introduction of exotic species.

In Mexico there are approximately 4111 insular marine structures, including oceanic islands, reefs, and cays (INEGI 2015). The geographical position, geological history, and ecological processes of this country allow its insular environments to have different climates and a great heterogeneity of habitats, where a high diversity of species with different evolutionary and biogeographic histories is established, many of which are endemic (Yáñez‐Arancibia et al. 2004; CONABIO‐CONANP‐TNC‐Pronatura 2007; Vilchis et al. 2018). Around 50% of the Mexican insular structures are found in the Mexican Atlantic, comprised of the Gulf of Mexico and Mexican Caribbean, whose transitory zone is at the north of the Yucatan Peninsula, where one of the country's reefs with the greatest biodiversity is located (Alacranes reef) (CONABIO‐CONANP‐TNC‐Pronatura 2007; Floeter et al. 2008; Tapia‐Silva et al. 2015; Vilchis et al. 2018).

One of the most representative groups of the insular environments of the Mexican Atlantic are the red algae, of which there is a lot of specific information about their geographical distribution (Dreckmann 1998; Ortegón‐Aznar and León‐Tejera 2022; Pedroche and Sentíes 2020). However, although these data have been compiled in different works (Dreckmann 1998; Littler and Littler 2000; Ortega et al. 2001; García‐García et al. 2020), the biodiversity and their patterns of distribution of these organisms in insular environments have not yet been analyzed, which could result in the detection of potentially important areas for the conservation of these organisms, which probably coincide with the Protected Natural Areas enacted for Mexico, and where algae have not been sufficiently considered.

In this study, our aim was to analyze the diversity and distribution of red algae from insular environments in the Mexican Atlantic to identify and describe their patterns of taxonomic diversity, priority areas for conservation, and the biogeographical affinities.

## 2 Material and Methods

### 2.1 Taxonomic Diversity

**FIGURE 1:**
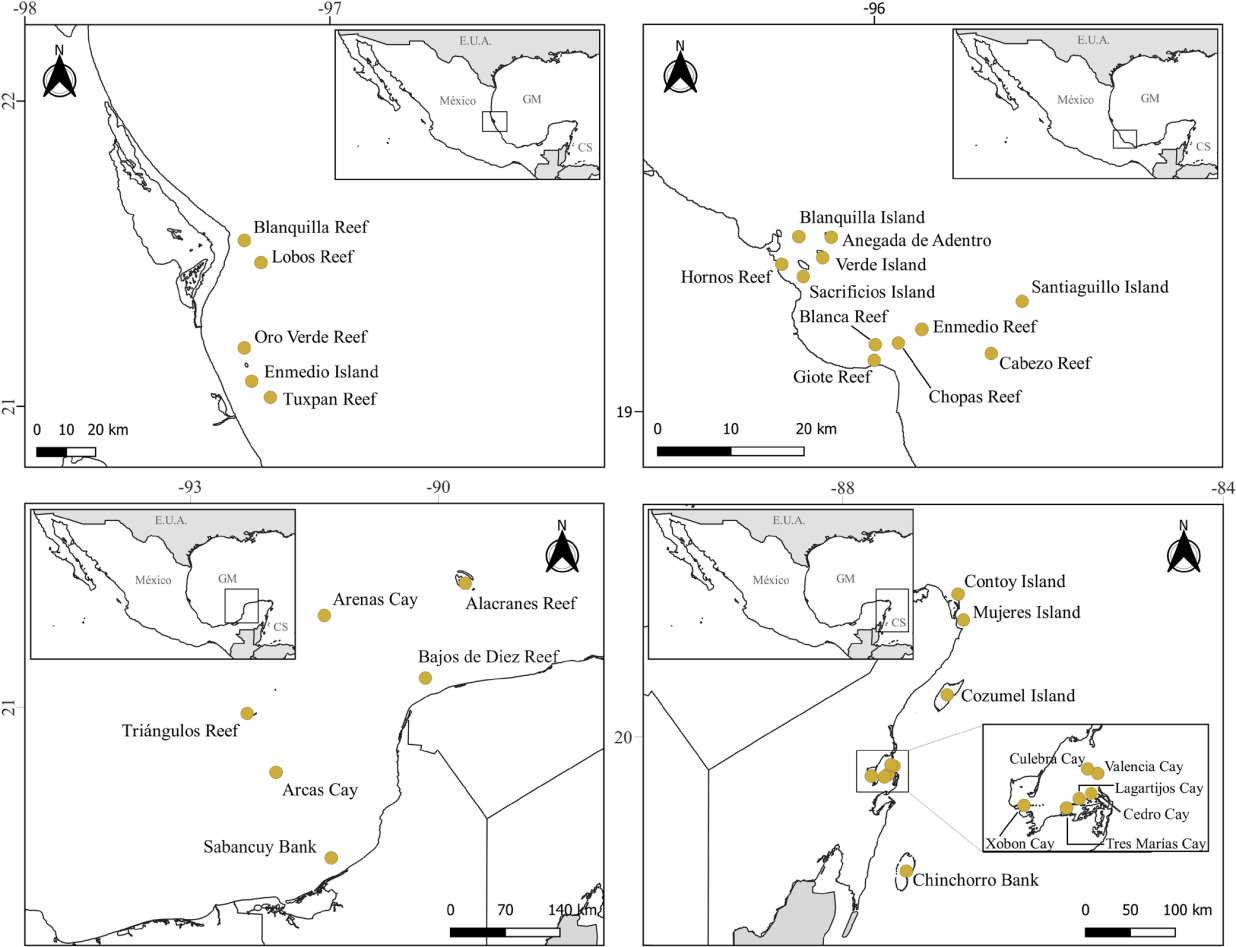
Location of the reef systems analyzed in this study. (A) Lobos‐Tuxpan Reef System, (B) Veracruz Reef System, (C) Campeche Bank Reef System and (D) Mesoamerican Reef System. CS, Caribbean Sea; GM, Gulf of Mexico.

A database with the species of red algae that have been recorded in insular environments from the Gulf of Mexico and the Mexican Caribbean (Mexican Atlantic) was assembled (Figure 1). The data were compiled from the red algae taxonomic update published by García‐García et al. (2020), complementing them with the information presented by Mendoza‐González and Mateo‐Cid (1985), Huerta‐Múzquiz et al. (1987), Mateo‐Cid and Mendoza‐González (1991), Mendoza‐González and Mateo‐Cid (1992), Mateo‐Cid et al. (1996), Dreckmann (1998), Littler and Littler (2000), Díaz‐Martín and Quan‐Young (2001), Ortega et al. (2001), Mateo‐Cid et al. (2002), De la Garza‐Flores (2003), Mateo‐Cid, Mendoza‐Gonzalez, and Searles (2006), Mateo‐Cid et al. (2007), Mendoza‐González et al. (2007), Cetz‐Navarro et al. (2008), Mendoza González et al. (2009), Galicia‐García et al. (2013), García‐López et al. (2013), Mateo‐Cid, Mendoza‐González, and Gabrielson (2014), Mateo‐Cid, Mendoza‐González, and García‐López (2014), Mendoza‐González et al. (2014), Mateo‐Cid et al. (2016), Luna Ortega and de la Cruz‐Francisco (2017), Pedroche and Sentíes (2020), de la Cruz Francisco et al. (2020), Mendoza‐González et al. (2024), Vilchis et al. (2024). Some records from Alacranes reef were obtained from Universidad Autónoma de Yucatán (UADY, México) macroalgae databases, and we present them for the first time in this publication. Each name and its taxonomic validity were revised in Algaebase database (Guiry and Guiry 2025). We also verified that all the insular environments analyzed were included in the catalog of Mexican insular territory published by INEGI (2015).

Based on the information from the database, the number of species, genera, families, and orders of each insular element were recorded to determine its taxonomic diversity. The insular structures analyzed belong to four reef systems (Figure 1): Lobos‐Tuxpan Reef System (SAL‐T, for its acronym in Spanish), Veracruz Reef System (SAV, for its acronym in Spanish), Campeche Bank Reef System (SABC, for its acronym in Spanish), and Mesoamerican Reef System (SAM, for its acronym in Spanish), all of them with distinct ecological conditions, geographical characteristics, and geological origin; therefore, the taxonomic diversity of each one was also recorded, and these study units were considered in the rest of the analyses.

The floristic similarity was evaluated by making a species presence‐absence matrix in each insular element and another similar matrix but for each reef system. The similarity among insular structures of the same reef system was calculated using the Jaccard similarity index, while for the comparisons among reef systems, a UPGMA (Unweighted Pair‐Group Method with Arithmetic Mean) cluster analysis was performed under the same index, which summarized the results in a dendrogram. Both analyses were performed in the software PAST v4.7b (Hammer et al. 2001).

### 2.2 Conservation

For the conservation proposal we considered the results of taxonomic richness, and a complementary analysis was carried out; this allows us to give conservation priority to a couple of areas that together contain the greatest number of species possible, being the first area the one with the greatest species richness and the second one that has the number of additional species not contained in the first, in other words, its complement (Álvarez‐Mondragón and Morrone 2004). This analysis was based on the index proposed by Colwell and Coddington (1994), which quantifies the dissimilarity in the composition of species between two areas using the equation:

$$\mathrm{ICC}:\left(A+B-2j\right)/\left(A+B-j\right)$$

where *A*: number of species in area 1, *B*: number of species in area 2, *j*: number of species in both areas. The value of the complementary index varies between 0 and 1; if its value is equal to 0, it indicates that they are areas with the same species (not complementary). When the value is equal to 1, the areas do not have species in common, so they are complementary to each other. This analysis was carried out considering all insular structures and among elements within each reef system.

It is important to highlight that the ICC can be affected when comparing insular systems composed of elements with very distant specific richness; in other words, very diverse elements and others with very poor diversity. In this case, the low number of species on certain islands causes a bias in trying to conserve as many species as possible, since many of these, having low richness, will surely be the complement of those other poor elements in their specific richness. The solution that we gave to this problem was to eliminate those insular structures with low diversity and thus preserve the greatest number of species.

### 2.3 Biogeographic Affinities

To know the biogeographic affinities of the species recorded in this study, their worldwide distribution was consulted in the Algaebase database (Guiry and Guiry 2025), which allowed us to classify them as follows:

#### 2.3.1 Disjunct

Species recorded in more than one ocean and whose area of distribution is fragmented by large geographic barriers.

#### 2.3.2 Amphiatlantic

Species that are distributed throughout the Atlantic Ocean.

#### 2.3.3 Western Atlantic

Species are distributed only on the America's Atlantic coast. Within this category we established three mutually inclusive subcategories: *Along Western Atlantic*, species distributed throughout the western Atlantic coast; *American Tropical Atlantic*, species whose distribution is restricted to the tropical zone of the western Atlantic (Gulf of Mexico and Caribbean Sea); *Caribbean Sea*, only Caribbean species.

## 3 Results

### 3.1 Taxonomic Diversity

345 taxonomically accepted species of red algae were reported in 32 insular environments of the Mexican Atlantic, which are included in 22 orders, 50 families and 148 genera (Appendix S1). The best represented families were Rhodomelaceae, Ceramiaceae and Delesseriaceae with 75, 34 and 27 species, respectively. Of all the species, 46 are reported exclusively in insular environments (Table 1). According to the information retrieved from the databases of the Universidad Autónoma de Yucatán, which is presented for the first time in this study, we report two new records for Mexico, specifically for Alacranes reef in Yucatán: *Lophosiphonia bermudensis* Collins & Hervey and *Polysiphonia saccorhiza* (Collins & Hervey) Hollenberg.

**TABLE 1:** Checklist of species reported only in insular environments of the Mexican Atlantic and their biogeographical affinities.

Species	Insular distribution in the Mexican Atlantic	Biogeographic affinity
*Acrochaetium antillarum* W.R. Taylor	Quintana Roo: Chinchorro bank	Western Atlantic
*Acrochaetium globosum* Børgesen	Quintana Roo: Cozumel Island	Disjunct (Atlantic, Indian, and Pacific oceans)
*Aglaothamnion uruguayense* (W.R. Taylor) N.E. Aponte, D.L. Ballantine & J.N. Norris	Quintana Roo: Cozumel Island	Western Atlantic
*Gaillona seposita* (Gunnerus) Athanasiadis	Quintana Roo: Mujeres Island	Amphiatlantic
*Gymnothamnion elegans* (Schousboe ex C. Agardh) J. Agardh	Yucatán: Alacranes reef; Quintana Roo: Cozumel Island	Disjunct (Atlantic, Indian, and Pacific oceans)
*Antithamnion antillanum* Børgesen	Quintana Roo: Cozumel Island	Disjunct (Atlantic, Indian, and Pacific oceans)
*Antithamnionella breviramosa* (E.Y. Dawson) Wollaston	Quintana Roo: Chinchorro bank	Disjunct (Atlantic, Indian, and Pacific oceans)
*Centrocerocolax ubatubensis* A.B. Joly	Quintana Roo: Cozumel Island	Amphiatlantic
*Ceramium dawsonii* A.B. Joly	Quintana Roo: Mujeres Island	Amphiatlantic
*Ceramium tenuicorne* (Kützing) Waern	Quintana Roo: Cozumel Island, Mujeres Island	Amphiatlantic
*Ceramium uruguayense* W.R. Taylor	Quintana Roo: Chinchorro bank	Amphiatlantic
*Pseudoceramium tenerrimum* (G. Martens) Barros‐Barreto & Maggs	Quintana Roo: Cozumel Island	Disjunct (Atlantic, Indian, and Pacific oceans)
*Dasya haitiana* S. Fredericq & J.N. Norris	Quintana Roo: Cozumel Island	Western Atlantic
*Heterosiphonia crispella* (C. Agardh) M.J. Wynne	Quintana Roo: Chinchorro Bank, Cozumel Island, Mujeres Island	Disjunct (Atlantic, Indian, and Pacific oceans)
*Hypoglossum simulans* M.J. Wynne, I.R. Price & D.L. Ballantine	Quintana Roo: Cozumel Island	Disjunct (Atlantic, Indian, and Pacific oceans)
*Nitophyllum wilkinsoniae* Collins & Hervey	Quintana Roo: Cozumel Island	Western Atlantic
*Thuretia bornetii* Vickers	Quintana Roo: Cozumel Island, Mujeres Island	Western Atlantic
*Chondria floridana* (Collins) M. Howe	Yucatán: Cayo Arenas, Alacranes reef; Quintana Roo: Chinchorro bank, Mujeres Island	Western Atlantic
*Chondrophycus anabeliae* Sentíes, M.T. Fujii, Cassano & Dreckmann	Quintana Roo: Mujeres Island	Western Atlantic
*Herposiphonia bipinnata* M. Howe	Quintana Roo: Cozumel Island	Western Atlantic
*Laurencia brongniartii* J. Agardh	Quintana Roo: Mujeres Island	Amphiatlantic
*Laurenciella marilzae* (Gil‐Rodríguez, Sentíes, Díaz‐Larrea, Cassano & M.T.Fujii) Gil‐Rodríguez, Sentíes, Díaz‐Larrea, Cassano & M.T.Fujii	Quintana Roo: Mujeres Island	Amphiatlantic
*Ohelopapa flexilis* (Setchell) F. Rousseau, Martin‐Lescanne, Payri & L. Le Gall	Quintana Roo: Cozumel Island	Disjunct (Atlantic, Indian, and Pacific oceans)
*Polysiphonia decusata* Hollenberg	Yucatán: Alacranes reef	Disjunct (Atlantic, Indian, and Pacific oceans)
*Yuzurua iridescens* (M.J. Wynne & D.L. Ballantine) Sentíes & M.J. Wynne	Quintana Roo: Mujeres Island	Western Atlantic
*Griffithsia schousboei* Montagne	Quintana Roo: Cozumel Island, Mujeres Island	Disjunct (Atlantic, Indian, and Pacific oceans)
*Haloplegma duperreyi* Montagne	Quintana Roo: Cozumel Island, Mujeres Island	Disjunct (Atlantic, Indian, and Pacific oceans)
*Spermothamnion macromeres* Collins & Hervey	Yucatán: Alacranes reef; Quintana Roo: Cozumel Island	Western Atlantic
*Colaconema robustum* (Børgesen) Huisman & Woelkerling	Yucatán: Cayo Arenas	Disjunct (Atlantic, Indian, and Pacific oceans)
*Goniolithon decutescens* (Heydrich) Foslie ex M. Howe	Veracruz: Isla Verde; Quintana Roo: Cozumel Island, Mujeres Island	Western Atlantic
*Lithophyllum incrustans* Philippi	Quintana Roo: Chinchorro bank	Disjunct (Atlantic and Indian oceans)
*Lithoporella atlantica* (Foslie) Foslie	Yucatán: Alacranes reef; Mujeres Island	Western Atlantic
*Lithoporella bermudensis* (Foslie) W.H. Adey	Quintana Roo: Cozumel Island, Mujeres Island	Western Atlantic
*Harveylithon munitum* (Foslie & M. Howe) A. Rösler, Perfectti, V. Peña & J.C. Braga	Yucatán: Alacranes reef; Quintana Roo: Cozumel Island, Mujeres Island	Disjunct (Atlantic and Indian oceans)
*Pterocladiella caloglossoides* (M. Howe) Santelices	Quintana Roo: Cozumel Island	(Atlantic, Indian, and Pacific oceans)
*Dudresnaya puertoricensis* Searles & D.L. Ballantine	Quintana Roo: Cozumel Island	Western Atlantic
*Chondracanthus elegans* (Greville) Guiry	Quintana Roo: Cozumel Island	Western Atlantic
*Gracilaria cuneata* Areschoug	Quintana Roo: Cozumel Island, Mujeres Island	Western Atlantic
*Gracilaria ornata* J.E. Areschoug	Quintana Roo: Mujeres Island	Western Atlantic
*Gracilaria wrightii* (Turner) J. Agardh	Quintana Roo: Cozumel Island, Mujeres Island	Disjunct (Atlantic, Indian, and Pacific oceans)
*Helminthocladia calvadosii* (J.V. Lamouroux ex Duby) Setchell	Quintana Roo: Cozumel Island	Western Atlantic
*Liagora tsengii* Huisman & M.J. Wynne	Quintana Roo: Cozumel Island	Western Atlantic
*Agissea simulans* (Weber Bosse) Pestana, Lyra, Cassano & J.M.C. Nunes	Quintana Roo: Cozumel Island, Mujeres Island	Disjunct (Atlantic, Indian, and Pacific oceans)
*Champia minuscula* A.B. Joly & Ugadim	Quintana Roo: Cozumel Island	Western Atlantic
*Asteromenia peltata* (W.R. Taylor) Huisman & A.J.K. Millar	Quintana Roo: Cozumel Island	Disjunct (Atlantic, Indian, and Pacific oceans)
*Cordylecladia peasiae* Collins	Quintana Roo: Mujeres Island	Western Atlantic

Cozumel Island, in Quintana Roo, was the insular environment with the greatest specific richness, followed by Mujeres Island, Chinchorro bank, and Alacranes reef. Similarly, these presented the greatest taxonomic diversity (Table 2).

**TABLE 2:** Taxonomic diversity of red algae in the 33 insular structures where there is at least one record of red algae.

State	Insular environment	Orders (%)	Families (%)	Genus (%)	Species (%)
Veracruz	Blanquilla reef	6 (27.2)	13 (26)	25 (16.8)	31 (9)
	Lobos reef	8 (36.3)	18 (36)	29 (19.5)	37 (10.7)
	Oro Verde reef	7 (31.8)	13 (26)	19 (12.8)	26 (7.5)
	Enmedio Island	10 (45.4)	23 (46)	46 (31.1)	71 (20.5)
	Tuxpan reef	8 (36.3)	15 (30)	24 (16.2)	32 (9.2)
	Blanquilla Island	5 (22.7)	10 (20)	12 (8.1)	15 (4.3)
	Anegada de Adentro	1 (4.5)	3 (6)	3 (2)	5 (1.4)
	Verde Island	11 (50)	21 (42)	37 (25)	52 (15)
	Hornos reef	9 (40.9)	14 (28)	24 (16.2)	35 (10.1)
	Sacrificios Island	9 (40.9)	16 (32.)	33 (22.2)	46 (13.3)
	Blanca reef	4 (18.8)	6 (12)	6 (4)	6 (1.7)
	Enmedio reef	10 (45.4)	21 (42)	36 (24.3)	46 (13.3)
	Giote reef	5 (22.7)	7 (14)	8 (5.4)	9 (2.6)
	Santiaguillo Island	6 (27.2)	13 (26)	20 (13.5)	25 (7.2)
	Cabezo reef	7 (31.8)	12 (24)	16 (10.8)	21 (6.1)
	Chopas reef	1 (4.5)	2 (4)	2 (1.3)	5 (1.4)
Campeche	Sabancuy bank	3 (13.6)	4 (8)	4 (2.7)	5 (1.4)
	Arcas cay	7 (31.8)	15 (30)	25 (16.8)	32 (9.2)
	Triángulos reef	7 (31.8)	14 (28)	20 (13.5)	24 (6.9)
Yucatán	Arenas cay	10 (45.4)	16 (32)	28 (18.9)	37 (10.7)
	Bajos de Diez reef	1 (4.5)	1 (2)	1 (0.6)	1 (0.3)
	Alacraes reef	12 (54.5)	27 (54)	60 (40.5)	114 (32.9)
Quintana Roo	Contoy Island	9 (40.9)	15 (30)	24 (16.2)	28 (8.1)
	Mujeres Island	17 (77.2)	37 (74)	104 (70.2)	208 (60.1)
	Cozumel Island	19 (86.3)	46 (98)	119 (80.4)	237 (68.5)
	Culebra cay	3 (13.6)	3 (6)	8 (5.4)	10 (2.9)
	Valencia cay	9 (40.9)	17 (34)	37 (25)	54 (15.6)
	Xobon cay	4 (18.1)	8 (16)	12 (8.1)	12 (3.5)
	Lagartijos cay	4 (18.1)	9 (18)	19 (12.8)	26 (7.5)
	Tres Marías cay	5 (22.7)	11 (22)	24 (16.2)	32 (9.2)
	Cedro cay	6 (27.2)	13 (26)	23 (15.5)	31 (9)
	Chinchorro bank	14 (63.6)	25 (50)	59 (39.8)	97 (28)

**FIGURE 2:**
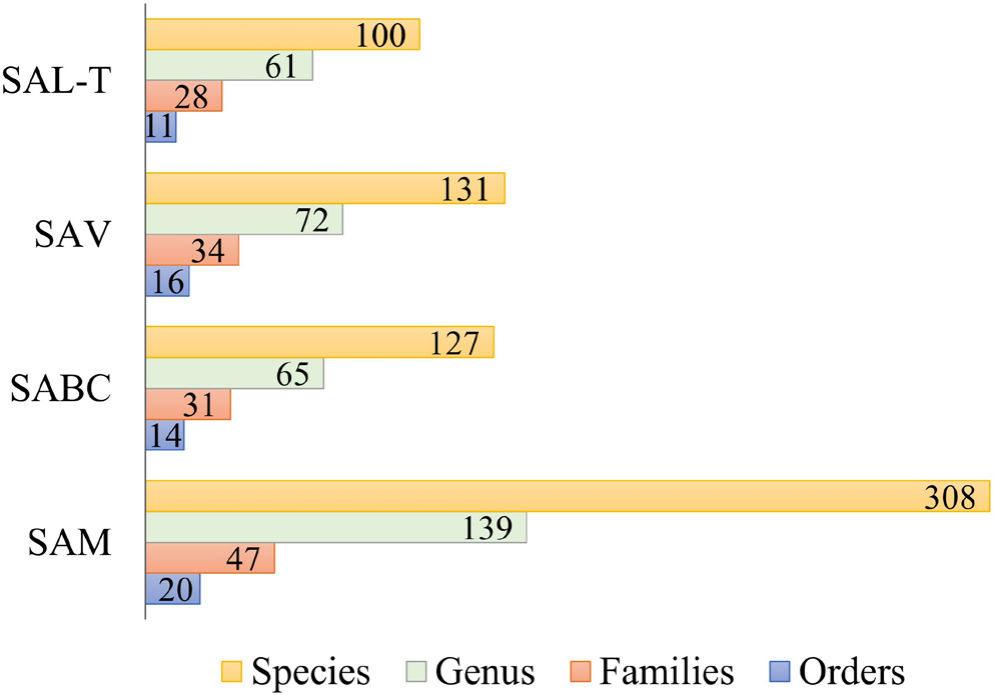
Taxonomic diversity of each reef system. Bars indicate the number of species, genus, families, and orders, as applicable.

In reference to the reef systems, the predominant families in all of them were Ceramiaceae and Rhodomelaceae (Table 3). The SAM was the one with the greatest species richness and taxonomic diversity, followed by the Campeche bank (Figure 2).

**TABLE 3:** Number of species per family in each archipelago.

Family/archipelago	Number of species per family			
	SAL‐T	SAV	SABC	SAM
Acrochaetiaceae	—	1	—	5
Acrosymphytaceae	—	—	—	1
Bangiaceae	—	1	1	1
Bonnemaisoniaceae	1	1	1	1
Callithamniaceae	4	3	5	14
Caulacanthaceae	—	—	—	2
Ceramiaceae	10	10	10	28
Champiaceae	2	3	3	4
Colaconemateaceae	1	1	1	3
Corallinaceae	4	5	5	6
Cystocloniaceae	3	4	2	4
Delesseriaceae	3	3	5	26
Dumontiaceae	1	—	—	2
Erythrotrichiaceae	2	2	3	3
Faucheaceae	—	—	—	1
Galaxauraceae	6	4	2	4
Gelidiaceae	2	3	2	5
Gelidiellaceae	2	2	2	3
Gigartinaceae	—	1	—	1
Gracilariaceae	1	9	7	19
Grateloupiaceae	—	—	—	1
Halymeniaceae	—	—	1	4
Hapalidiaceae	2	3	2	4
Hildenbrandiaceae	—	1	—	—
Hydrolithaceae	3	3	3	4
Hymenocladiaceae	1	1	—	1
Kallymeniaceae	—	—	—	1
Liagoraceae	5	8	8	10
Lithophyllaceae	10	10	6	10
Lomentariaceae	2	3	1	3
Mastophoraceae	—	—	1	2
Mesophyllaceae	—	1	—	1
Naccariaceae	—	—	—	1
Nemastomataceae	1	1	—	1
Nemaliaceae	1	—	—	—
Peyssonneliaceae	—	—	1	9
Phyllophoraceae	—	—	—	2
Porolithaceae	2	3	2	4
Pterocladiaceae	2	2	3	4
Rhizophyllidaceae	—	—	1	1
Rhodochaetaceae	—	1	1	2
Rhodogorgonaceae	—	1	—	—
Rhodomelaceae	21	24	29	65
Rhodymeniaceae	—	1	1	8
Scinaiaceae	—	—	—	1
Sebdeniaceae	—	—	—	1
Solieriaceae	1	3	1	6
Spongitaceae	4	8	9	12
Stylonemataceae	—	2	2	2
Wrangeliaceae	3	2	6	15

The floristic similarity among insular structures within each reef system ranged as follows: from 15% to 40% for the SAL‐T, from 0% to 66% for the SAV, from 0% to 56% SABC and from 0% to 60% for the SAM. The greatest floristic similarity was found for the following island pairs: Tuxpan reef—Lobos reef (SAL‐T, 40%), Chopas reef–Anegada de Adentro reef (SAV, 66%), Arcas cay—Arenas cay (SABC, 56%), and Mujeres Island—Cozumel Island (SAM, 60%).

**FIGURE 3:**
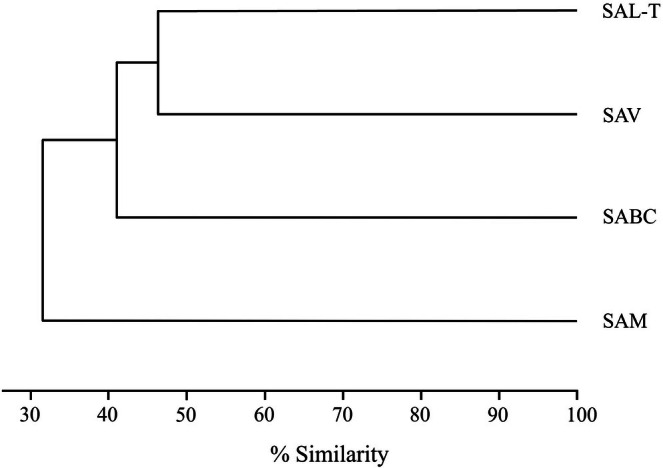
Dendrogram of floristic similarity among the studied reef systems.

According to the dendrogram, all reef systems share a certain floristic composition; however, similarity values are low. The most similar reef systems were SAV and SAL‐T (Figure 3).

### 3.2 Conservation

The complementarity analysis shows that Cozumel Island (Quintana Roo) and Anegada de Adentro reef (Veracruz) are the insular structures that complement each other the best, presenting an ICC of 0.98, which indicates that 98% of their specific composition is different, allowing the preservation of 238 out of the 345 registered insular red algae species in these two areas in the Mexican Atlantic.

Within SAL‐T, Oro Verde reef and Enmedio island were the areas that showed the highest complementarity value (ICC = 0.73), with a 73% difference in their specific composition. With the protection of this pair of areas, 71 out of the 100 registered red algae species of this reef system would be conserved.

Regarding the SAV, most pairwise comparisons resulted in an ICC = 1. The low number of species present in half of its islands makes them the ideal complement to those with higher richness, preventing the confident conservation of the greatest number of species possible in a pair of areas. Consequently, we decided to exclude these elements from the analysis, and as a result, Verde Island and Giote reef constitute the pair of islands that, together, host the highest possible specific richness (ICC = 0.96), enabling the conservation of 59 out of the 131 species present in this reef system.

In the Campeche Bank, the highest complementarity was observed between Alacranes reef and Sabancuy Bank with an ICC = 0.96; therefore, both areas differ almost entirely in their phycofloristic composition. The preservation of this pair of areas will allow the protection of 115 out of the 127 species of red algae recorded in this insular environment system.

Finally, in the SAM, Cozumel Island and Culebra cay showed the greatest complementarity, with an ICC = 0.93, and together they contain 231 species of red algae out of the 308 recorded for the area.

### 3.3 Biogeographic Affinities

Of the 345 species listed in this study, 243 have a disjunct distribution, 18 are Amphiatlantic, and 84 are only present in the Western Atlantic; of these last, 19 are distributed in the tropical zone of the Western Atlantic, while 43 are found in the Caribbean Sea.

**FIGURE 4:**
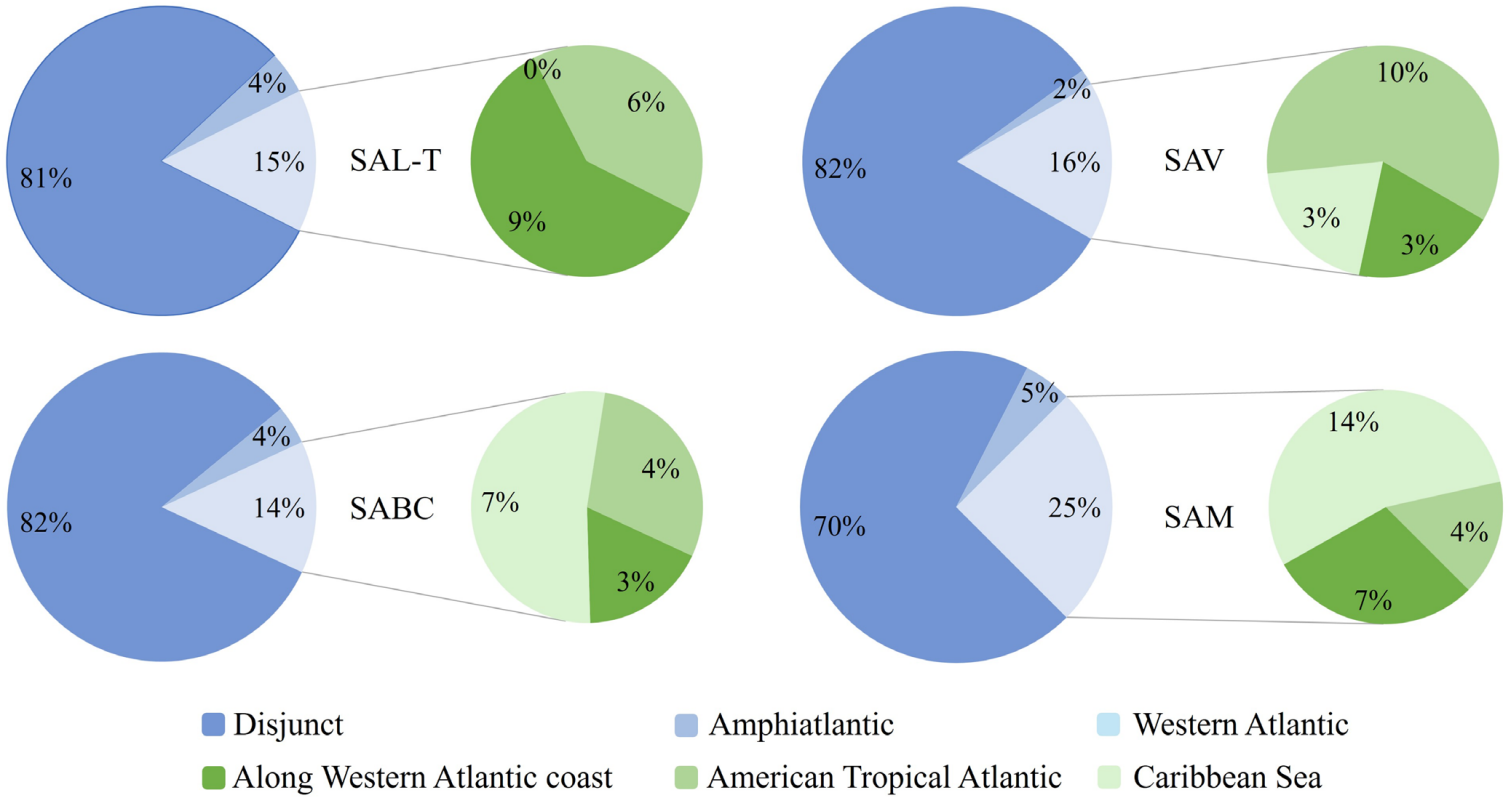
Biogeographic affinities of the 345 insular species recorded in this study represented as percentages.

In all reef systems, disjunct species predominated, followed by species with an affinity for the Western Atlantic, and finally, the Amphiatlantic species. Regarding the subcategories established within the western Atlantic, the predominant insular red algae in the SAV, SABC, and SAM were those with affinities to the American Tropical Atlantic, while in the SAL‐T, those with affinities to the Caribbean Sea predominated (Figure 4).

## 4 Discussion

### 4.1 Taxonomic Diversity

In Mexican Atlantic, the best represented red algal families are Rhodomelaceae, Ceramiaceae, Delesseriaceae and Gracilariaceae, the first three are included in the order Ceramiales and consist of 96, 43 and 34 species, respectively, while the last belongs to the order Gracilariales and has 34 recorded species (Dreckmann 1998; Pedroche and Sentíes 2020; García‐García et al. 2020; Carranza‐Ramírez et al. 2023; Vilchis et al. 2024). Our results indicate that the diversity of insular environments does not differ, highlighting that from the total species included in each mentioned family, there are those reported exclusively in these environments, which represented 16% for Ceramiaceae, 14% for Delesseriaceae, 10% for Rhodomelaceae and 9% for Gracilariaceae. The evolutionary and ecological success of Ceramiales is not only reflected in their high diversity, which worldwide accounts for one‐third of all red algae (Díaz‐Tapia et al. 2019), but also in their wide distribution, with occurrences in cold, temperate and tropical regions (Guiry and Guiry 2025). Their components have adapted to different marine environments resulting in a wide variety of forms, from the simplest filaments to arborescent or laminar thalli, with specialized structures that enable them to adhere to various substrates and resist forces generated by water movement. Another important factor contributing to this success is their reproductive processes, both sexual and asexual, with the former involving the release of spores into the environment, where they remain dormant until favorable conditions occur; and the latter, in which, despite being almost entirely detached by water movement, a single stipe can continue to divide and regenerate the thallus (Hernández et al. 2021).

According to the floristic list present in this study, Cozumel Island, Alacranes reef, and Mujeres Island were the most taxonomically diverse insular structures (Table 2). This is consistent because these are the largest among the sites analyzed, with 468, 250, and 182 km^2^, respectively, which supports the premise that insular species richness increases with insular structures area and further increases with their proximity to the continent (MacArthur and Wilson 1967; Lomolino and Weiser 2001; Rodríguez and Vázquez‐Domínguez 2003). These insular structures contribute significantly to making the reef systems of the Yucatan Peninsula the most diverse in the Mexican Atlantic. This general pattern of diversity has not only been reported for other groups of macroalgae (Tapia‐Silva et al. 2015; Núñez Resendiz et al. 2016; Vilchis et al. 2018), but also for organisms such as annelids (Granados Barba et al. 2003), mollusks (Escobar 2004; Castillo‐Rodríguez 2014), fish (Floeter et al. 2008), echinoids (Caballero‐Ochoa et al. 2017), and macrocrustaceans (Escobar‐Briones and Jiménez‐Guadarrama 2010). Vilchis et al. (2018) explain this pattern of diversity as the result of the geological evolution of the region; this resulted in the fragmentation of ancestral areas and biotas, which led to the formation of the current biotic components of the Gulf of Mexico and the Mexican Caribbean, whose convergence in the northwestern part of the Yucatan Peninsula is considered a biogeographic and ecological transition zone.

On the other hand, floristic similarity values were low among the insular structures within each reef system (except for the SAV), and considerably lower among different reef systems. This is likely related to the high habitat heterogeneity characteristic of the area, where spatial variation could be influencing the establishment and proliferation of macroalgae, many of which are adapted to specific microenvironments, which leads to notable differences in the biological composition of nearby localities, promoting high species richness (MacArthur and MacArthur 1961; Cramer and Willig 2005; Pedroche and Sentíes 2020; Vilchis et al. 2024).

### 4.2 Conservation

In Mexico, conservation efforts have primarily focused on protecting areas where conspicuous species, those of economic or cultural importance, or species that are listed under some category of risk (CONABIO‐CONANP‐TNC‐Pronatura 2007). Marine macroalgae are not included in any of these criteria, and therefore, have been protected indirectly. Vilchis et al. (2018) suggested for the first time five priority areas for the conservation of these organisms from Mexican Atlantic, which encompassed the entire coast of the Yucatan Peninsula and part of Veracruz, However, the size of these areas is very large, which could make protecting them difficult due to the associated costs and resource requirements. Our proposal aimed to maximize species protection within a limited number of areas. Cozumel Island (Quintana Roo) and Anegada de Adentro reef (Veracruz) were identified as the highest priority sites, protecting these two sites would allow for the conservation of 238 of the 345 red algal species recorded in this study, this represents 69% of the insular diversity and 51% of the total diversity of rhodophytes present in the Gulf of Mexico and the Mexican Caribbean. Within each reef system, the total percentage of species protected by pairs of insular structures ranged from 69% to 98%, indicating a substantial difference in their species composition, which is related, like the similarity values, to the biotic heterogeneity of the region. The results strengthen the criteria established by Vilchis et al. (2018) and highlight the overlap of these areas with several priority sites for the conservation of marine biodiversity, as proposed by CONABIO‐CONANP‐TNC‐Pronatura (2007).

### 4.3 Biogeographic Affinities

Regarding biogeographic affinities, 70% of the recorded species have a disjunct distribution range, while the remaining 30% have a restricted distribution range. According to Díaz‐Tapia et al. (2018), disjunct distribution is due to three main reasons: (1) invasive or introduced species, whose distribution tends to be very widespread, often becoming cosmopolitan species; (2) cryptic species complex, whose morphological similarity masks different taxonomic identities, most of which have a more restricted distribution than previously thought; (3) widely distributed species with strong phylogeographic structure. Specifically, for red algae, there are few confirmed cases of invasive or introduced species, but three clear examples are *Acanthophora spicifera* (Vahl) Børgesen (De Jong et al. 1999; Aguilar‐Rosas et al. 2014), *Asparagopsis taxiformis* (Delile) Trevisan (Nahor et al. 2022) and *Kappaphycus alvarezii* (Doty) L.M. Liao (Ask et al. 2003). On the other hand, one of the main challenges in the macroalgae group is the presence of cryptic species, since historically, their diversity has been based on morphological attributes, which have begun to be clarified using molecular tools, resulting in substantial changes in the classification of these organisms (Núñez Resendiz et al. 2024). However, according to a compilation by Núñez Resendiz et al. (2024), of the 1700 species of red, green and brown algae reported for Mexico, the taxonomic identity of only 13% has been confirmed, further hindering the precise definition of their biogeographic affinities. An example is the case of *Digenea* C. Agardh, since the morphological monotony among its members led to it being considered a monotypic genus for over 50 years; currently, six additional species have been described based on molecular evidence, all of them with more restricted distribution and distinct biogeographic affinities (Schneider et al. 2018; Boo et al. 2018; Soares et al. 2022; Vilchis et al. 2022). Some similar examples are the species *Gracilariopsis lemaneiformis* (Bory) E.Y. Dawson, Acleto & Foldvik (Hernandez et al. 2020), *Gracilaria domingensis* complex (Lyra et al. 2016), *Laurencia obtusa* (Hudson) J.V. Lamouroux (Cassano et al. 2012) and *Palisada perforata* (Bory) K.W. Nam (Cassano et al. 2009; Sentíes et al. 2009), as well as some genera such as *Centroceras* Kützing (Won et al. 2009), *Gelidiella* Feldmann & G. Hamel (Núñez Resendiz et al. 2023) and *Hypnea* J.V. Lamouroux (Nauer et al. 2015; de Jesus et al. 2016).

About 30% of species exhibited restricted distributions, some have already been confirmed through molecular evidence, which allowed us to determine their biogeographic affinities with greater certainty, either Western Atlantic or Amphiatlantic. Concerning the subcategories attributed to the Western Atlantic, slightly more than 50% exhibit affinity with the Caribbean Sea, with the Mesoamerican Reef System showing the highest proportion of species with this affinity, and this deceases toward the north of the country, with no occurrence in the Lobos‐Tuxpan Reef System, where subtropical (temperate) macroalgae are predominant, and it has also been identified as a transition zone between tropical and temperate marine floras (Ortega et al. 2001), reflecting the convergence of two biogeographic realms: Temperate Northern Atlantic and Tropical Atlantic (Spalding et al. 2007).

Each aspect analyzed in this study highlights the diversity of red algae present in the insular environments of the Mexican Atlantic, as well as their composite nature, shaped by biotas of different biogeographic origins; this has resulted in a mosaic of species with distinct biogeographic affinities. The above provides clear evidence of the importance of continuing conservation efforts in areas where these organisms are present, as they are currently not included in protection regulations despite their conspicuous nature and the important ecological functions they perform across virtually all marine ecosystems.

## Author Contributions

**Martha Isabel Vilchis:** conceptualization (lead), data curation (equal), formal analysis (equal), investigation (equal), methodology (lead), software (equal), supervision (equal), writing – original draft (equal), writing – review and editing (lead). **Oscar E. Hernández:** conceptualization (lead), data curation (equal), formal analysis (equal), methodology (equal), software (equal), supervision (equal), writing – original draft (equal), writing – review and editing (equal). **María Luisa Núñez Resendiz:** data curation (equal), formal analysis (supporting), writing – review and editing (equal). **Kurt M. Dreckmann:** supervision (equal), writing – review and editing (equal). **Ileana Ortegón‐Aznar:** data curation (supporting), project administration (equal), supervision (equal), writing – review and editing (equal). **Abel Sentíes:** supervision (equal), writing – review and editing (equal).

## Conflicts of Interest

The authors declare no conflicts of interest.

## Supporting information


**Appendix S1:** ece372919‐sup‐0001‐AppendixS1.docx.

**Data S1:** ece372919‐sup‐0002‐DataS1.xls.

## Data Availability

The data that supports the findings of the study are available as Supporting Information.
