# Supplementary material for: Red Algae From Insular Environments in the Mexican Atlantic: Taxonomic Diversity, Conservation, and Biogeographic Affinities
Source: Ecol Evol. 2026 Mar 17;16(3):e72919. doi: 10.1002/ece3.72919 (PMC13093592; doi:10.1002/ece3.72919)
Supplement: Supplementary file 1 — Appendix S1: ece372919‐sup‐0001‐AppendixS1.docx. [file ECE3-16-e72919-s002.docx]

**Appendix 1.** List of red algae recorded in the present study, organized according to the classification system of Wynne (2022).

| **Order** | **Family** | **Species** |
| --- | --- | --- |
| Bangiales | Bangiaceae | *Bangia atropurpurea* (Mertens ex Roth) C. Agardh |
| Acrochaetiales | Acrochaetiaceae | *Acrochaetium antillarum* W.R. Taylor |
|  |  | *Acrochaetium flexuosum* Vickers |
|  |  | *Acrochaetium globosum* Børgesen |
|  |  | *Acrochaetium microscopicum* (Nägeli ex Kützing) Nägeli |
|  |  | *Acrochaetium unipes* Børgesen |
| Acrosymphytales | Acrosymphytaceae | *Acrosymphyton caribaeum* (J. Agardh) G. Sjöstedt |
| Bonnemaisoniales | Bonnemaisoniaceae | *Asparagopsis taxiformis* (Delile) Trevisan |
|  | Naccariaceae | *Naccaria antillana* W.R. Taylor |
| Ceramiales | Callithamniaceae | *Aglaothamnion boergesenii* (Aponte & D.L. Ballantine) L'Hardy-Halos & Rueness |
|  |  | *Aglaothamnion cordatum* (Børgesen) Feldmann-Mazoyer |
|  |  | *Aglaothamnion felipponei* (Howe) Aponte, Ballantine & J.N. Norris |
|  |  | *Aglaothamnion halliae* (Collins) Aponte, D.L. Ballantine & J.N. Norris |
|  |  | *Aglaothamnion uruguayense* (W.R. Taylor) N.E. Aponte, D.L. Ballantine & J.N. Norris |
|  |  | *Callithamnion corymbosum* (Smith) Lyngbye |
|  |  | *Crouania attenuata* (C. Agardh) J. Agardh |
|  |  | *Crouania mayae* Mateo-Cid, Mendoza-González & Searles |
|  |  | *Crouania pleonospora* W.R. Taylor |
|  |  | *Gaillona seposita* (Gunnerus) Athanasiadis |
|  |  | *Gymnothamnion elegans* (Schousboe ex C. Agardh) J. Agardh |
|  |  | *Seirospora occidentalis* Børgesen |
|  |  | *Spyridia filamentosa* (Wulfen) Harvey |
|  |  | *Spyridia hypnoides* (Bory) Papenfuss |
|  | Ceramiaceae | *Acrothamnion butlerae* (Collins) Kylin |
|  |  | *Antithamnion antillanum* Børgesen |
|  |  | *Antithamnion cruciatum* (C. Agardh) Nägeli |
|  |  | *Antithamnion decipiens* (J. Agardh) Athanasiadis |
|  |  | *Antithamnionella boergesenii* (Cormaci & G. Furnari) Athanasiadis |
|  |  | *Antithamnionella breviramosa* (E.Y. Dawson) E.M. Wollaston |
|  |  | *Callithamniella tingitana* (Schousboe ex Bornet) Feldmann-Mazoyer |
|  |  | *Centroceras clavulatum* (C. Agardh) Montagne |
|  |  | *Centroceras gasparrinii* (Meneghini) Kützing |
|  |  | *Centroceras micracanthum* Kützing |
|  |  | *Centrocerocolax ubatubensis* A.B. Joly |
|  |  | *Ceramium brasiliense* A.B. Joly |
|  |  | *Ceramium caudatum* Setchell & N.L. Gardner |
|  |  | *Ceramium cimbricum* H.E. Petersen |
|  |  | *Ceramium codii* (H. Richards) Mazoyer |
|  |  | *Ceramium corniculatum* Montagne |
|  |  | *Ceramium cruciatum* Collins & Hervey |
|  |  | *Ceramium deslongchampsii* Chauvin ex Duby |
|  |  | *Ceramium diaphanum* (Lightfoot) Roth |
|  |  | *Ceramium leptozonum* M. Howe |
|  |  | *Ceramium luetzelburgii* O.C. Schmidt |
|  |  | *Ceramium nitens* (C. Agardh) J. Agardh |
|  |  | *Ceramium subtile* J. Agardh |
|  |  | *Ceramium tenuicorne* (Kützing) Wærn |
|  |  | *Ceramium uruguayense* W.R. Taylor |
|  |  | *Ceramium virgatum* Roth |
|  |  | *Dohrniella antillarum* (W.R. Taylor) Feldmann-Mazoyer |
|  |  | *Gayliella flaccida* (Harvey ex Kützing) T.O. Cho & L.M. McIvor |
|  |  | *Gayliella dawsonii* (A.B. Joly) Barros-Barreto & F.P. Gomes |
|  |  | *Gayliella mazoyerae* T.O. Cho, Fredericq & Hommersand |
|  |  | *Pseudoceramium tenerrimum* (G. Martens) Barros-Barreto & Maggs |
|  |  | *Pseudoceramium brevizonatum* (H.E. Petersen) Barros-Barreto & Maggs |
|  |  | *Yoneshiguea compta* (Børgesen) Barros-Barreto, Maggs & M.A. Jaramillo |
|  | Delesseriaceae | *Caloglossa leprieurii* (Montagne) G. Martens |
|  |  | *Dasya caraibica* Børgesen |
|  |  | *Dasya corymbifera* J. Agardh |
|  |  | *Dasya crouaniana* J. Agardh |
|  |  | *Dasya haitiana* S. Fredericq & J.N. Norris |
|  |  | *Dasya hutchinsiae* Harvey |
|  |  | *Dasya mollis* Harvey |
|  |  | *Dasya ocellata* (Grateloup) Harvey |
|  |  | *Dasya pedicellata* (C. Agardh) C. Agardh |
|  |  | *Dasya ramosissima* Harvey |
|  |  | *Dasya rigidula* (Kützing) Ardissone |
|  |  | *Dasysiphonia collinsiana*(M. Howe) M.M. Cassidy, C.W. Schneider & G.W. Saunders |
|  |  | *Dictyurus occidentalis* J. Agardh |
|  |  | *Halydictyon mirabile* Zanardini |
|  |  | *Heterosiphonia crispella* (C. Agardh) M.J. Wynne |
|  |  | *Heterosiphonia gibbesii* (Harvey) Falkenberg |
|  |  | *Hypoglossum hypoglossoides* (Stackhouse) Collins & Hervey |
|  |  | *Hypoglossum simulans* M.J. Wynne, I.R. Price & D. L. Ballantine |
|  |  | *Hypoglossum subsimplex* M.J. Wynne |
|  |  | *Hypoglossum tenuifolium* (Harvey) J. Agardh |
|  |  | *Martensia fragilis* Harvey |
|  |  | *Martensia pavonia* (J. Agardh) J. Agardh |
|  |  | *Nitophyllum adhaerens* M.J. Wynne |
|  |  | *Nitophyllum wilkinsoniae* Collins & Hervey |
|  |  | *Taenioma nanum* (Kützing) Papenfuss |
|  |  | *Taenioma perpusillum* (J. Agardh) J. Agardh |
|  |  | *Thuretia bornetii* Vickers |
|  | Rhodomelaceae | *Acanthophora muscoides* (Linnaeus) Bory |
|  |  | *Acanthophora spicifera* (Vahl) Børgesen |
|  |  | *Acanthosiphonia echinata* (Harvey) Savoie & G.W. Saunders |
|  |  | *Alsidium seaforthii* (Turner) J. Agardh |
|  |  | *Alsidium triquetrum* (S.G. Gmelin) Trevisan |
|  |  | *Amansia multífida* J.V. Lamouroux |
|  |  | *Bostrychia binderi* Harvey |
|  |  | *Bostrychia montagnei* Harvey |
|  |  | *Bostrychia radicans* (Montagne) Montagne |
|  |  | *Bostrychia scorpioides* (Hudson) Montagne |
|  |  | *Bostrychia tenella* (J. V. Lamouroux) J. Agardh |
|  |  | *Bryocladia atlantica*(Kapraun & J.N. Norris) Díaz-Tapia |
|  |  | *Bryocladia cuspidata* (J. Agardh) De Toni |
|  |  | *Bryocladia subtilissima*(Montagne) Díaz-Tapia |
|  |  | *Bryocladia villum*(J. Agardh) Díaz-Tapia |
|  |  | *Carradoriella denudata* (Dillwyn) Savoie & G.W. Saunders |
|  |  | *Chondria atropurpurea*Harvey |
|  |  | *Chondria baileyana* (Montagne) Harvey |
|  |  | *Chondria capillaris* (Hudson) M. J. Wynne |
|  |  | *Chondria cnicophylla* (Melvill) De Toni |
|  |  | *Chondria collinsiana* M. Howe |
|  |  | *Chondria curvilineata* Collins & Hervey |
|  |  | *Chondria collinsiana* M. Howe |
|  |  | *Chondria dasyphylla* (Woodward) C. Agardh |
|  |  | *Chondria floridana* (Collins) M.Howe |
|  |  | *Chondria leptacremon* (Melvill ex G. Murray) De Toni |
|  |  | *Chondria littoralis* Harvey |
|  |  | *Chondria platyramea* A. B. Joly & Ugadim |
|  |  | *Chondria polyrhiza* Collins & Hervey |
|  |  | *Chondria sedifolia* Harvey |
|  |  | *Chondrophycus anabeliae* Sentíes, M.T. Fujii, Cassano & Dreckmann |
|  |  | *Digenea mexicana* G.H.Boo & D. Robledo |
|  |  | *Dipterosiphonia dendritica* (C. Agardh) F. Schmitz |
|  |  | *Dipterosiphonia rigens* (C. Agardh) Falkenberg |
|  |  | *Herposiphonia bipinnata* M. Howe |
|  |  | *Herposiphonia pecten-veneris (*Harvey) Falkenberg |
|  |  | *Herposiphonia secunda* (C. Agardh) Ambronn |
|  |  | *Herposiphonia tenella* (C. Agardh) Ambronn |
|  |  | *Heterodasya mucronata* (Harvey) M.J. Wynne |
|  |  | *Laurencia brongniartii* J. Agardh |
|  |  | *Laurencia caraibica* P.C. Silva |
|  |  | *Laurencia filiformis* (C. Agardh) Montagne |
|  |  | *Laurencia intricata* J.V. Lamouroux |
|  |  | *Laurencia microcladia* Kützing |
|  |  | *Laurencia obtusa* (Hudson) J.V. Lamouroux |
|  |  | *Laurenciella marilzae*(Gil-Rodríguez, Sentíes, Díaz-Larrea, Cassano & M.T. Fujii) Gil-Rodríguez, Sentíes, Díaz-Larrea, Cassano & M.T. Fujii |
|  |  | *Lophocladia trichoclados* (C. Agardh) F. Schmitz |
|  |  | *Lophosiphonia bermudensis* Collins & Hervey |
|  |  | *Lophosiphonia cristata* Falkenberg |
|  |  | *Lophosiphonia obscura* (C. Agardh) Falkenberg |
|  |  | *Melanothamnus ferulaceus* (Suhr ex J. Agardh) Díaz-Tapia & Maggs |
|  |  | *Melanothamnus gorgoniae* (Harvey) Díaz-Tapia & Maggs |
|  |  | *Melanothamnus pseudovillum* (Hollenberg) Díaz-Tapia & Maggs |
|  |  | *Melanothamnus sphaerocarpus* (Børgesen) Díaz-Tapia & Maggs |
|  |  | *Murrayella periclados* (C. Agardh) F. Schmitz |
|  |  | *Ohelopapa flexilis* (Setchell) F. Rousseau, Martin-Lescanne, Payri & L. Le Gall |
|  |  | *Ophidocladus simpliciusculus* (P. Crouan & H. Crouan) Falkenberg |
|  |  | *Osmundaria obtusiloba* (C. Agardh) R.E. Norris |
|  |  | *Palisada corallopsis* (Montagne) Sentíes, M.T. Fujii & Díaz-Larrea |
|  |  | *Palisada flagellifera* (J. Agardh) K.W. Nam |
|  |  | *Palisada perforata* (Bory) K.W. Nam |
|  |  | *Polysiphonia binneyi* Harvey |
|  |  | *Polysiphonia breviarticulata* (C. Agardh) Zanardini |
|  |  | *Polysiphonia decusata* Hollenberg |
|  |  | *Polysiphonia exilis* Harvey |
|  |  | *Polysiphonia havanensis* Montagne |
|  |  | *Polysiphonia saccorhiza* (Collins & Hervey) Hollenberg |
|  |  | *Polysiphonia sertularioides* (Grateloup) J. Agardh |
|  |  | *Wilsonosiphonia howei*(Hollenberg) D. Bustamante, Won & T.O. Cho |
|  |  | *Wrightiella blodgettii* (Harvey) F.Schmitz |
|  |  | *Wrightiella tumanowiczii* (Gatty ex Harvey) F. Schmitz |
|  |  | *Yuzurua iridescens* (M.J. Wynne & D.L. Ballantine) Sentíes & M.J. Wynne |
|  |  | *Yuzurua poiteaui* (J.V. Lamouroux) Martin-Lescanne |
|  | Wrangeliaceae | *Anotrichium tenue* (C. Agardh) Nägeli |
|  |  | *Griffithsia globulifera* Harvey ex Kützing |
|  |  | *Griffithsia heteromorpha* Kützing |
|  |  | *Griffithsia radicans* Kützing |
|  |  | *Griffithsia schousboei* Montagne |
|  |  | *Haloplegma duperreyi* Montagne |
|  |  | *Ptilothamnion speluncarum* (Collins & Hervey) D.L. Ballantine & M.J. Wynne |
|  |  | *Spermothamnion gymnocarpum* M. Howe |
|  |  | *Spermothamnion investiens* (P. Crouan & H. Crouan) Vickers |
|  |  | *Spermothamnion macromeres* Collins & Hervey |
|  |  | *Spermothamnion repens* (Dillwyn) Magnus |
|  |  | *Tiffaniella gorgonea* (Montagne) Doty & Meñez |
|  |  | *Wrangelia argus* (Montagne) Montagne |
|  |  | *Wrangelia bicuspidata* Børgesen |
|  |  | *Wrangelia penicillata* (C. Agardh) C. Agardh |
| Colaconematales | Colaconemateaceae | *Colaconema daviesii* (Dillwyn) Stegenga |
|  |  | *Colaconema hallandicum* (Kylin) Afonso-Carrillo, Sanson, Sangil & Diaz-Villa |
|  |  | *Colaconema hypneae* (Børgesen) A.A. Santos & C.W.N. Moura |
|  |  | *Colaconema robustum* (Børgesen) Huisman & Woelkerling |
| Corallinales | Corallinaceae | *Jania capillacea* Harvey |
|  |  | *Jania cubensis* Montagne ex Kützing |
|  |  | *Jania pedunculata* J.V. Lamouroux |
|  |  | *Jania pumila* J.V. Lamouroux |
|  |  | *Jania rubens (Linnaeus)* J.V. Lamouroux |
|  |  | *Jania subulata* (J. Ellis & Solander) Sonder |
|  | Hydrolithaceae | *Hydrolithon boergesenii* (Foslie) Foslie |
|  |  | *Hydrolithon farinosum* (J.V. Lamouroux) Penrose & Y.M. Chamberlain |
|  |  | *Pneophyllum confervicola* (Kützing) Y.M. Chamberlain |
|  |  | *Pneophyllum fragile* Kützing |
|  | Lithophylloideae | *Amphiroa beauvoisii* J.V. Lamouroux |
|  |  | *Amphiroa brasiliana* Decaisne |
|  |  | *Amphiroa fragilissima* (Linnaeus) J.V. Lamouroux |
|  |  | *Amphiroa hancockii* W.R. Taylor |
|  |  | *Amphiroa rígida* J.V. Lamouroux |
|  |  | *Amphiroa tribulus* (J. Ellis & Solander) J.V. Lamouroux |
|  |  | *Amphiroa vanbosseae* Me. Lemoine |
|  |  | *Goniolithon decutescens* (Heydrich) Foslie ex M. Howe |
|  |  | *Lithophyllum corallinae* (P. Crouan & H. Crouan) Heydrich |
|  |  | *Lithophyllum incrustans* Philippi |
|  |  | *Lithophyllum intermedium* Foslie |
|  |  | *Lithophyllum kaiseri* (Heydrich) Heydrich |
|  |  | *Lithophyllum prototypum* (Foslie) Foslie |
|  |  | *Lithophyllum stictiforme* (Areschoug) Hauck |
|  |  | *Titanoderma pustulatum* (J.V. Lamouroux) Nägeli |
|  | Mastophoraceae | *Lithoporella atlantica* (Foslie) Foslie |
|  |  | *Lithoporella bermudensis* (Foslie) W.H. Adey |
|  | Porolithaceae | *Harveylithon munitum* (Foslie & M. Howe) A. Rösler, Perfectti, V. Peña & J.C. Braga |
|  |  | *Porolithon antillarum* (Foslie & M. Howe) Foslie & M. Howe |
|  |  | *Porolithon improcerum* (Foslie & M.Howe) M. Howe |
|  |  | *Porolithon onkodes* (Heydrich) Foslie |
|  | Spongitidaceae | *Neogoniolithon acropetum* (Foslie & M. Howe) W.H. Adey |
|  |  | *Neogoniolithon erosum* (Foslie) W.H. Adey |
|  |  | *Neogoniolithon fosliei* (Heydrich) Setchell & L.R. Mason |
|  |  | *Neogoniolithon mamillare* (Harvey) Setchell & L.R. Mason |
|  |  | *Neogoniolithon propinquum* (Foslie) Me. Lemoine |
|  |  | *Neogoniolithon rhizophorae* (Foslie & M. Howe) Setchell & L.R. Mason |
|  |  | *Neogoniolithon siankanense*L.E. Mateo-Cid, A.C. Mendoza-González & P.W. Gabrielson |
|  |  | *Neogoniolithon solubile* (Foslie & M. Howe) Setchell & L.R. Mason |
|  |  | *Neogoniolithon spectabile* (Foslie) Setchell & L.R. Mason |
|  |  | *Neogoniolithon strictum* (Foslie) Setchell & L.R. Mason |
|  |  | *Neogoniolithon trichotomum* (Heydrich) Setchell & L.R. Mason |
|  |  | *Spongites absimilis* (Foslie & M. Howe) Afonso-Carrillo |
|  |  | *Spongites yendoi* (Foslie) Y.M. Chamberlain |
| Gelidiales | Gelidiellaceae | *Gelidiella acerosa* (Forsskål) Feldmann & Hamel |
|  |  | *Gelidiella lubrica* (Kützing) Feldmann & Hamel |
|  |  | *Millerella pannosa* (Feldmann) G.H. Boo & L. Le Gall |
|  |  | *Parviphycus trinitatensis* (W.R. Taylor) M.J. Wynne |
|  | Gelidiaceae | *Gelidium americanum* (W.R. Taylor) Santelices |
|  |  | *Gelidium corneum* (Hudson) J.V. Lamouroux |
|  |  | *Gelidium crinale* (Hare ex Turner) Gaillon |
|  |  | *Gelidium pusillum* (Stackhouse) Le Jolis |
|  |  | *Gelidium spinosum* (S.G. Gmelin) P.C. Silva |
|  | Pterocladiaceae | *Pterocladiella caerulescens* (Kützing) Santelices & Hommersand |
|  |  | *Pterocladiella bartlettii* (W.R. Taylor) Santelices |
|  |  | *Pterocladiella caloglossoides* (M. Howe) Santelices |
|  |  | *Pterocladiella capillacea* (S.G. Gmelin) Santelices & Hommersand |
|  |  | *Pterocladiella sanctarum* (Feldmann & Hamel) Santelices |
| Gigartinales | Caulacanthaceae | *Catenella caespitosa* (Withering) L.M. Irvine |
|  |  | *Catenella impudica* (Montagne) J. Agardh |
|  | Cystocloniaceae | *Hypnea cervicornis* J. Agardh |
|  |  | *Hypnea cornuta* (Kützing) J. Agardh |
|  |  | *Hypnea musciformis* (Wulfen) J.V. Lamouroux |
|  |  | *Hypnea spinella* (C. Agardh) Kützing |
|  |  | *Hypnea valentiae* (Turner) Montagne |
|  |  | *Hypneocolax stellaris* Børgesen |
|  | Dumontiaceae | *Dudresnaya crassa* M. Howe |
|  |  | *Dudresnaya puertoricensis* Searles & D.L. Ballantine |
|  | Gigartinaceae | *Chondracanthus acicularis* (Roth) Fredericq |
|  |  | *Chondracanthus elegans* (Greville) Guiry |
|  | Kallymeniaceae | *Austrokallymenia westii* (Ganesan) C.W. Schneider & G.W. Saunders |
|  | Phyllophoraceae | *Gymnogongrus griffithsiae* (Turner) Martius |
|  |  | *Gymnogongrus tenuis* J. Agardh |
|  | Rhizophyllidaceae | *Contarinia magdae* Weber Bosse |
|  |  | *Ochtodes secundiramea* (Montagne) M. Howe |
|  | Solieriaceae | *Agardhiella ramosissima* (Harvey) Kylin |
|  |  | *Agardhiella subulata* (C. Agardh) Kraft & M.J. Wynne |
|  |  | *Eucheumatopsis isiformis* (C. Agardh) Núñez-Resendiz, Dreckmann & Sentíes |
|  |  | *Flahaultia tegetiformans* W.R. Taylor |
|  |  | *Solieria filiformis* (Kützing) P.W. Gabrielson |
|  |  | *Wurdemannia miniata* (Sprengel) Feldmann & Hamel |
| Gracilariales | Gracilariaceae | *Gracilaria caudata* J. Agardh |
|  |  | *Gracilaria cornea* J. Agardh |
|  |  | *Gracilaria crassissima* (P. Crouan & H. Crouan) P. Crouan & H. Crouan |
|  |  | *Gracilaria usneoides* (C. Agardh) J. Agardh |
|  |  | *Gracilaria blodgettii* Harvey |
|  |  | *Gracilaria bursa-pastoris* (S.G. Gmelin) P.C. Silva |
|  |  | *Gracilaria cervicornis* J. Agardh |
|  |  | *Gracilaria cuneata* Areschoug |
|  |  | *Gracilaria cylindrica* Børgesen |
|  |  | *Gracilaria damicornis* J. Agardh |
|  |  | *Gracilaria debilis* (Forsskål) Børgesen |
|  |  | *Gracilaria domingensis* (Kützing) Sonder ex Dickie |
|  |  | *Gracilaria foliifera* (Forsskål) Børgesen |
|  |  | *Gracilaria gracilis* (Stackhouse) Steentoft, L.M. Irvine & Farnham |
|  |  | *Gracilaria mammillaris* (Montagne) M. Howe |
|  |  | *Gracilaria ornata* J.E. Areschoug |
|  |  | *Gracilaria wrightii* (Turner) J. Agardh |
|  |  | *Gracilariopsis longissima* (S.G. Gmelin) Steentoft, L.M. Irvine & Farnham |
|  |  | *Gracilariopsis tenuifrons* (C.J. Bird & E.C. Oliveira) Fredericq & Hommersand |
| Halymeniales | Grateloupiaceae | *Grateloupia filicina* (J.V. Lamouroux) C. Agardh |
|  | Halymeniaceae | *Corynomorpha clavata*(Harvey) J. Agardh |
|  |  | *Cryptonemia crenulata* (J. Agardh) J. Agardh |
|  |  | *Cryptonemia floridana*(J. Agardh) J.P. Soares, T. Sauvage & M.T. Fujii |
|  |  | *Halymenia pseudofloresii*Collins & M.Howe |
|  |  | *Halymenia rosea* M. Howe & W.R. Taylor |
| Hapalidiales | Hapalidiaceae | *Melobesia membranacea*(Esper) J.V. Lamouroux |
|  |  | *Lithothamnion occidentale* (Foslie) Foslie |
|  |  | *Lithothamnion sejunctum* Foslie |
|  |  | *Roseolithon crispatum* (Hauck) P.W. Gabrielson, Maneveldt, Hughey & V. Peña |
|  | Mesophyllaceae | *Mesophyllum mesomorphum* (Foslie) W.H. Adey |
| Hildenbrandiales | Hildenbrandiaceae | *Hildenbrandia rubra* (Sommerfelt) Meneghini |
| Nemaliales | Galaxauraceae | *Dichotomaria marginata* (J. Ellis & Solander) Lamarck |
|  |  | *Dichotomaria obtusata* (J. Ellis & Solander) Lamarck |
|  |  | *Dichotomaria marginata* (J. Ellis & Solander) Lamarck |
|  |  | *Galaxaura rugosa* (J. Ellis & Solander) J.V. Lamouroux |
|  |  | *Tricleocarpa cylindrica* (J. Ellis & Solander) Huisman & Borowitzka |
|  |  | *Tricleocarpa fragilis* (Linnaeus) Huisman & R.A. Townsend |
|  | Liagoraceae | *Ganonema farinosum* (J.V. Lamouroux) K.-C. Fan & Yung C. Wang |
|  |  | *Ganonema megagynum* (Børgesen) Huisman |
|  |  | *Ganonema pinnatum* (Harvey) Huisman |
|  |  | *Gloiocallis dendroidea* (P. Crouan & H. Crouan) Showe M. Lin, Huisman & D.L. Ballantine |
|  |  | *Helminthocladia calvadosii* (J.V. Lamouroux ex Duby) Setchell |
|  |  | *Liagora albicans* J.V. Lamouroux |
|  |  | *Liagora ceranoides* J.V. Lamouroux |
|  |  | *Liagora tsengii* Huisman & M.J. Wynne |
|  |  | *Titanophycus validus* (Harvey) Huisman, G.W. Saunders & A.R. Sherwood |
|  |  | *Trichogloea herveyi* W.R. Taylor |
|  |  | *Trichogloea requienii* (Montagne) Kützing |
|  |  | *Trichogloeopsis pedicellata* (M.Howe) I.A. Abbott & Doty |
|  | Nemaliaceae | *Nemalion elminthoides* (Velley) Batters |
|  | Scinaiaceae | *Scinaia complanata* (Collins) Cotton |
| Nemastomatales | Nemastomataceae | *Predaea feldmannii* Børgesen |
| Peyssonneliales | Peyssonneliaceae | *Agissea inamoena*(Pilger) Pestana, Lyra, Cassano & J.M.C. Nunes |
|  |  | *Agissea simulans*(Weber Bosse) Pestana, Lyra, Cassano & J.M.C. Nunes |
|  |  | *Agissea stoechas*(Boudouresque & Denizot) Pestana, Lyra, Cassano & J.M.C.Nunes |
|  |  | *Olokunia boudouresquei*(Yoneshigue) Pestana, Lyra, Cassano & J.M.C. Nunes |
|  |  | *Rhodowynnea nordstedtii*(Weber Bosse) Pestana, Lyra, Cassano & J.M.C. Nunes |
|  |  | *Peyssonnelia armorica* (P. Crouan & H. Crouan) Weber Bosse |
|  |  | *Peyssonnelia boergesenii* Weber Bosse |
|  |  | *Peyssonnelia conchicola* Piccone & Grunow |
|  |  | *Peyssonnelia rubra* (Greville) J. Agardh |
| Rhodymeniales | Champiaceae | *Champia minuscula* A.B. Joly & Ugadim |
|  |  | *Champia parvula* (C. Agardh) Harvey |
|  |  | *Champia salicornioides* Harvey |
|  |  | *Coelothrix irregularis* (Harvey) Børgesen |
|  |  | *Gastroclonium parvum* (Hollenberg) C.F. Chang & B.M. Xi |
|  | Faucheaceae | *Gloioderma atlanticum* Searles |
|  | Hymenocladiaceae | *Asteromenia peltata* (W.R. Taylor) Huisman & A. Millar |
|  | Lomentariaceae | *Ceratodictyon intricatum* (C. Agardh) R.E. Norris |
|  |  | *Ceratodictyon planicaule* (W.R. Taylor) M.J. Wynne |
|  |  | *Ceratodictyon variable* (J. Agardh) R.E. Norris |
|  |  | *Lomentaria rawitscheri* A.B. Joly |
|  | Rhodymeniaceae | *Botryocladia enteromorpha* (Harvey) W.E. Schmidt, Lozada-Troche, D.L. Ballantine & Fredericq |
|  |  | *Botryocladia occidentalis* (Børgesen) Kylin |
|  |  | *Botryocladia pyriformis* (Børgesen) Kylin |
|  |  | *Botryocladia spinulifera* W.R. Taylor & I.A. Abbott |
|  |  | *Chrysymenia halymenioides* Harvey |
|  |  | *Coelarthrum cliftonii* (Harvey) Kylin |
|  |  | *Cordylecladia peasiae* Collins |
|  |  | *Rhodymenia pseudopalmata* (J.V. Lamouroux) P.C. Silva |
| Rhodogorgonales | Rhodogorgonaceae | *Rhodogorgon ramosissima* J.N. Norris & Bucher |
| Sebdeniales | Sebdeniaceae | *Sebdenia flabellata* (J. Agardh) P.G. Parkinson |
| Erythropeltales | Erythrotrichiaceae | *Erythrocladia irregularis* Rosenvinge |
|  |  | *Erythrocladia pinnata* W.R. Taylor |
|  |  | *Erythrotrichia carnea* (Dillwyn) J. Agardh |
|  |  | *Sahlingia subintegra* (Rosenvinge) Kornmann |
| Rhodochaetales | Rhodochaetaceae | *Rhodochaete pulchella* Thuret ex Bornet |
| Stylonematales | Stylonemataceae | *Chroodactylon ornatum* (C. Agardh) Basson |
|  |  | *Stylonema alsidii* (Zanardini) K.M. Drew |
